# Antibiotics in Wastewater of a Rural and an Urban Hospital before and after Wastewater Treatment, and the Relationship with Antibiotic Use—A One Year Study from Vietnam

**DOI:** 10.3390/ijerph13060588

**Published:** 2016-06-14

**Authors:** La Thi Quynh Lien, Nguyen Quynh Hoa, Nguyen Thi Kim Chuc, Nguyen Thi Minh Thoa, Ho Dang Phuc, Vishal Diwan, Nguyen Thanh Dat, Ashok J. Tamhankar, Cecilia Stålsby Lundborg

**Affiliations:** 1 Global Health—Health Systems and Policy (HSP), Medicines, Focusing Antibiotics, Department of Public Health Sciences, Karolinska Institutet, Tomtebodavägen 18 A, Stockholm 17177, Sweden; vishaldiwan@hotmail.com (V.D.); ejetee@gmail.com (A.J.T.); Cecilia.Stalsby.Lundborg@ki.se (C.S.L.); 2 Department of Pharmaceutical Management and Pharmaco-Economics, Hanoi University of Pharmacy, 13-15 Le Thanh Tong, Hoan Kiem District, Hanoi, Vietnam; 3 Department of Pharmacy, National Cancer Hospital, 30 Cau Buou, Thanh Tri District, Hanoi, Vietnam; quynhhoa29@gmail.com; 4 Department of Family Medicine, Hanoi Medical University, 01 Ton That Tung, Dong Da District, Hanoi, Vietnam; ntkchuc@yahoo.com (N.T.K.C.); ntmthoa@gmail.com (N.T.M.T.); 5 Institute of Mathematics-VAST, 18 Hoang Quoc Viet, Cau Giay District, Hanoi, Vietnam; hodang54@yahoo.com; 6 Department of Public Health & Environment, R.D. Gardi Medical College, Agar Road, Ujjain 456006, India; 7 Hanoi Drug and Cosmetic Testing Centre, 7/107 Nguyen Chi Thanh, Dong Da district, Hanoi, Vietnam; cnlname@yahoo.com; 8 Indian Initiative for Management of Antibiotic Resistance, Department of Environmental Medicine, R.D. Gardi Medical College, Agar Road, Ujjain 456006, India

**Keywords:** antibiotic residues, hospital wastewater, ciprofloxacin, metronidazole

## Abstract

Hospital effluents represent an important source for the release of antibiotics and antibiotic resistant bacteria into the environment. This study aims to determine concentrations of various antibiotics in wastewater before and after wastewater treatment in a rural hospital (60 km from the center of Hanoi) and in an urban hospital (in the center of Hanoi) in Vietnam, and it aims to explore the relationship between antibiotic concentrations in wastewater before wastewater treatment and quantities of antibiotics used in the rural hospital, over a period of one year in 2013. Water samples were collected using continuous sampling for 24 h in the last week of every month. The data on quantities of antibiotics delivered to all inpatient wards were collected from the Pharmacy department in the rural hospital. Solid-phase extraction and high performance liquid chromatography-tandem mass spectrometry were used for chemical analysis. Significant concentrations of antibiotics were present in the wastewater both before and after wastewater treatment of both the rural and the urban hospital. Ciprofloxacin was detected at the highest concentrations in the rural hospital’s wastewater (before treatment: mean = 42.8 µg/L; after treatment: mean = 21.5 µg/L). Metronidazole was detected at the highest concentrations in the urban hospital’s wastewater (before treatment: mean = 36.5 µg/L; after treatment: mean = 14.8 µg/L). A significant correlation between antibiotic concentrations in wastewater before treatment and quantities of antibiotics used in the rural hospital was found for ciprofloxacin (*r* = 0.78; *p* = 0.01) and metronidazole (*r* = 0.99; *p* < 0.001).

## 1. Introduction

The concern about antibiotic pollution of the environment in general, and of aquatic ecosystems in particular, is increasing globally [1,2,3]. Antibiotics are one of the most important medicines in hospitals and large amounts of antibiotics can be released into hospital wastewater due to excretion of used antibiotics and disposal of unused compounds. The occurrence of antibiotics in the aquatic environment might promote the selection of antibiotic resistance genes and antibiotic resistant bacteria [4]. Thus, hospital effluents represent an important source for the release of antibiotics and antibiotic resistant bacteria into the environment [5]. This exacerbates the situation of antibiotic resistance, which is an increasingly serious threat to global public health [6].

The occurrence of antibiotic residues in aquatic environments and hospital effluent has been studied, but mostly in higher income countries [1,2,5,7,8,9]. Studies are relatively sparse in low- and lower middle-income countries (LMICs) [10,11]. However, documentation of the situation in LMICs countries would be more important as in these countries hospital treatment plants either do not exist or do not function effectively. Literature has shown that quite rarely national legal regulations have been established to define how to treat hospital effluent [12]. In Vietnam, a lower middle-income country, the first regulation on healthcare waste management was issued in 1999 by the Ministry of Health. The current valid regulation was issued in 2007 [13]. According to both the regulations, every Vietnamese hospital must have a wastewater treatment plant. However, nearly 50% of Vietnamese hospitals across the country did still not have wastewater treatment plants in 2014 [14].

To the best of our knowledge, there has been only one study which investigated residual concentration of antibiotics in hospital wastewater in Vietnam. This study used grab sampling [11]. It is however suggested that continuous sampling should be the method of choice to determine antibiotic concentrations in hospital wastewater as grab sampling gives the quantities of antibiotics present in wastewater only at the time of sampling [15]. Moreover, very few studies explore the correlation between antibiotic residues in hospital wastewater and antibiotic use in the hospitals, which is important in developing the understanding of the relationship between antibiotic use and environmental reservoirs of antibiotics. Therefore, we conducted a study to (i) determine the concentrations of various antibiotics in wastewater before as well as after wastewater treatment using continuous sampling in a rural and an urban hospital in Hanoi, Vietnam; and (ii) explore the relationship between the antibiotic concentrations in wastewater before wastewater treatment and the quantities of antibiotics used in the rural hospital, over a period of one year in 2013.

## 2. Materials and Methods

### 2.1. Design

This was a prospective repeated cross-sectional study with data collection every month in 2013.

### 2.2. Setting

The study was conducted at two general hospitals in Hanoi: a rural and an urban hospital. Both the hospitals had wastewater treatment plants which were located on the hospital sites and treated only hospital wastewater. The rural hospital is situated in a rural setting, 60 km north-west of the center of Hanoi. The catchment area of the hospital is the district where the hospital is situated. It had 220 beds with the following departments: Anesthesia and Critical Care, Dermatology, Diagnostic Imaging, Infectious Diseases, Internal Medicine, Obstetrics, Odontology, Ophthalmology, Otolaryngology, Out-patients, Pediatrics, Pharmacy, Surgery, Testing, Traditional Medicines, and Intensive Care Unit. The wastewater treatment plant of the rural hospital was functioning using filtrations, biological treatment, and disinfection. The urban hospital is located in the center of Hanoi. The hospital patients are mainly from within the city. It has 520 beds with the following departments: Artificial Kidney Unit, Anesthesia and Critical Care, Biochemistry, Gastroenrerology, Hematology and Blood Tranfusion, Internal Medicines, Microbiology, Neuphro-Urology, Neurology, Neurosurgery, Obstetrics, Odontology, Oncology, Orthopaedics and Traumatology, Outpatients, Pathology, Pediatrics, Pharmacy, and Physiotherapy and Rehabilitation. The wastewater treatment plant of the urban hospital used physical, chemical, and biological treatment along with activated sludge and adhesive materials.

### 2.3. Selection of Antibiotics

Seven antibiotics from six antibiotic groups were selected for the study based on the antibiotic prescription pattern in the inpatient wards of the hospitals, the degree of metabolism of antibiotics in the human body and the known suspected environmental impact of an antibiotic [1,10]. The selected antibiotics are presented in the order: names, abbreviations, Anatomical Therapeutic Chemical (ATC) codes and antibiotic groups: (i) metronidazole (MET): J01XD01 (Imidazole derivatives); (ii) sulfamethoxazole (SUL): J01EC01 (Intermediate-acting sulfonamides); (iii) trimethoprim (TRI): J01EA01 (Trimethoprim and derivatives); (iv) ceftazidime (CEF): J01DD02 (Third-generation cephalosporins); (v) ciprofloxacin (CIP): J01MA02 (Fluoroquinolones); (vi) ofloxacin (OFL): J01MA01 (Fluoroquinolones); (vii) spiramycin (SPI): J01FA02 (Macrolides) [16].

### 2.4. Collection of Water Samples and Antibiotic Use Data

In each hospital, samples were taken from wastewater before as well as after wastewater treatment and from incoming water. Incoming water is water from a piped supply. It was tested to unequivocally prove that antibiotic residues in the hospital wastewater were from the hospital use of antibiotics only. Sampling of water was done on weekdays one time for 24 h using continuous sampling in the last week in every month in 2013. For every sampling event in both the hospitals, the following procedure was followed: samples of incoming water were taken from the water tanks, which supplied water to the hospitals. Wastewater was collected both before and after treatment continuously for 24 h using a pump with a flow speed of 40 drops/min connected with a closed container surrounded by ice and protected from light [15]. For each type of water, three liters of water was collected during 24 h. Water samples of 500 mL each for all three types of water were stored in amber colored glass bottles, wrapped in silver foil and clearly labeled. The water samples were placed in an ice box with a lid, protected from light, and reached the chemical laboratory of Hanoi Drug and Cosmetic Testing Center within 12 h for analysis. In the testing center, the samples remained at −20 °C until they were analyzed. The urban hospital, including its wastewater treatment plant, was under reconstruction from June to August 2013. Therefore, sampling could not be done in the hospital during this period. In total, there were 66 water samples including 12 samples of incoming water, 12 samples of wastewater before treatment, and 12 samples of wastewater after treatment from the rural hospital; and 12 samples of incoming water, 9 samples of wastewater before treatment, and 9 samples of wastewater after treatment from the urban hospital. In order to analyze the association, if any, between antibiotic concentrations in hospital wastewater and hospital antibiotic use, data on the quantities of antibiotics delivered to all inpatient wards were collected from the department of Pharmacy in the rural hospital. For the urban hospital this was not feasible. Hereafter, the quantities of antibiotics delivered to inpatient wards will be referred as the quantities of antibiotics used.

### 2.5. Water Sample Analysis

The quantitative determination of antibiotic concentrations of the seven studied antibiotics in hospital water was done following the method described by Diwan *et al.* [10]. In brief, a water sample was filtered through 0.45-µ-filter membrane and acidified with formic acid to pH 3.0, and then subjected to solid phase extraction for isolating analytes. The selected antibiotics were detected using high performance liquid chromatography-tandem mass spectrometry (Shimadzu LCMS-8030, Shimadzu Corporation, Kyoto, Japan). The antibiotics were separated on reverse-phase column (C18; 250 mm × 4.6 mm, 5 µm) which was operated at 30 °C at the flow rate of 0.5 mL/min. The mobile-phase solvents were water-acetonitrile (1:1) acidified with acetic acid to pH 3.0 for MET, SUL, TRI, CEF, CIP, and OFL, and acetonitrile-ammonium acetate (60:40) for SPI. The antibiotics were detected using a triple quadruple mass spectrometer equipped with electrospray ionization (positive-ion mode). The limit of detection (LOD) and limit of quantification (LOQ) of studied antibiotics (LOD; LOQ) were: MET (0.12 µg/L; 0.40 µg/L), SUL (0.003 µg/L; 0.009 µg/L), TRI (0.01 µg/L; 0.04 µg/L), CEF (0.011 µg/L; 0.036 µg/L), CIP (0.04 µg/L; 0.13 µg/L); OFL (0.03 µg/L; 0.1 µg/L); SPI (0.0001 µg/L; 0.0003 µg/L).

### 2.6. Data Management and Statistical Analysis

Data on antibiotic concentrations and the quantities of antibiotics used in the hospital were collected every month and entered in Microsoft Excel and then extracted into Stata 12 (StataCorp LP, College Station, TX, USA) for analysis. Antibiotic concentrations are presented in µg/L, while the quantities of antibiotics used is presented in grams (g/month). Descriptive statistics were used to present mean, minimum, and maximum values. The Wilcoxon signed rank test was applied to compare antibiotic concentrations in wastewater before and after wastewater treatment for each hospital. To determine the relationship between quantities of antibiotics used and antibiotic concentrations in hospital wastewater, Spearman’s correlation was performed. Correlations between the quantities of antibiotics used in one month and antibiotic concentrations in wastewater in the same month, after one, two, and three months were assessed.

## 3. Results

### 3.1. Antibiotic Residue Levels in Hospital Wastewater

No antibiotics were detected in any of the samples of incoming water from both the hospitals. Total concentrations of the studied antibiotics per month over a period of one year (*i.e.*, the concentration of each antibiotic from each sample added together) are presented in Table 1.

All seven studied antibiotics were detected in the wastewater of the rural hospital and six antibiotics were detected in the wastewater of the urban hospital both before and after wastewater treatment. Overall, in wastewater from both of the hospitals, the total concentration of studied antibiotics per month after treatment was lower than before treatment (rural hospital: *p* = 0.002; urban hospital: *p* = 0.008). The average difference in total concentrations of studied antibiotics per month after wastewater treatment for the rural and the urban hospitals were 49% and 67.3% respectively. In the rural hospital, 654.9 g of the studied antibiotics were used per month and 34.0 µg/L of the studied antibiotics were released per month into the environment. From the urban hospital, a total of 32.4 µg/L of the studied antibiotics were released per month into the environment.

In the rural hospital, among the seven studied antibiotics, CIP was detected at the highest concentration (Table 2). Another fluoroquinolone, OFL, was detected in the lowest concentrations. The antibiotic which was detected most rarely in water samples over the one year period was CEF. It was found only in water samples of two months, March and April. The average difference in concentrations of each studied antibiotic per month after wastewater treatment ranged from 35.2% to 80.5%. When comparing concentrations in wastewater before and after treatment for each studied antibiotic the differences were significant for MET, TRI, CIP, OFL ,and SPI (*p* = 0.01; 0.002; 0.002; 0.003; 0.004 respectively). There was no significant difference in case of SUL and CEF (*p* = 0.06; 0.16 respectively).

In the case of the urban hospital, MET was detected at the highest concentration among the studied antibiotics, followed by CIP (Table 3). CEF was not detected in any of the wastewater samples over the one year period. The average difference in concentrations of each studied antibiotic per month after wastewater treatment ranged from 51.3% to 79.6%. When comparing concentrations in wastewater before and after treatment for each studied antibiotic, the differences were significant for all the six detected antibiotics: MET, SUL, TRI, CIP, OFL, and SPI (*p* = 0.03; 0.02; 0.01; 0.008; 0.009; 0.04 respectively).

### 3.2. Relationship between Antibiotic Use and Antibiotic Residues in Hospital Wastewater of the Rural Hospital

**Figure 1:**
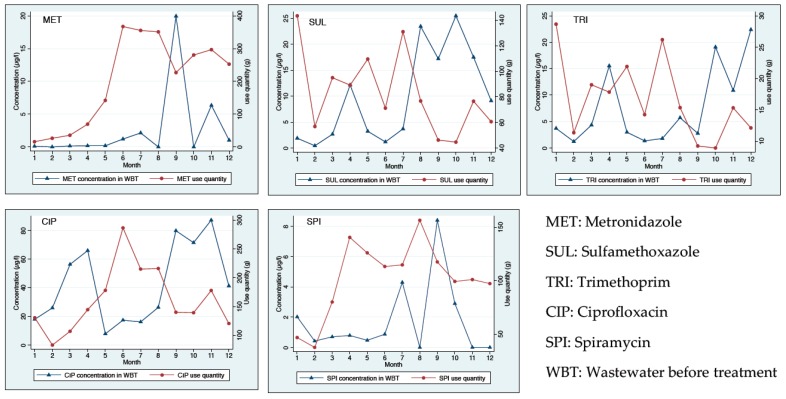
Relationship between quantities of antibiotics used and residues of studied antibiotics in wastewater before treatment of a rural hospital in Vietnam.

Spearman’s correlation was performed for MET, SUL, TRI, CIP, and SPI. It was not performed in the case of CEF and OFL because CEF was detected only in water samples of two months and OFL was not in the antibiotic use data of the hospital. Significant correlations were found between the quantities of antibiotics used in the rural hospital and antibiotic concentrations in wastewater before treatment in case of MET and CIP. The correlation between the quantities of MET used during six months (January to June) and the MET concentrations the month after use (February to July) was positive and high (*r* = 0.99; *p* < 0.001). The correlation between the quantities of CIP used during the nine months (January to September) and the CIP concentrations three months after use (April to December) was also positive and high (*r* = 0.78; *p* < 0.01). In the case of other antibiotics, no correlation was detected (Figure 1).

## 4. Discussion

To our knowledge, this is the first study which has attempted to identify, concurrently, antibiotic concentrations in hospital wastewater and its relationship with quantities of antibiotics used in the same hospital over a period of one year.

Our findings indicate that wastewater treatment plants in both the hospitals resulted in lower concentrations of the studied antibiotics after wastewater treatment as compared to before. However, significant concentrations of antibiotics were still present in the hospital effluents which, when released to the environment, could promote the selection of antibiotic resistant bacteria. The average difference in concentrations of CIP after wastewater treatment in this study (58.6% and 49.8% for the rural and the urban hospital, respectively) is less than in another study from another Vietnamese hospital (86%) [11]. In that study, water samples were collected during only two days using grab sampling, while in our study the water samples were collected every month over a period of one year using a continuous sampling system. The differences in concentrations of CIP after wastewater treatment in the present study are also relatively less compared with the figures in some high-income countries reported in other studies: 59%–76% in USA, 92% in Switzerland and 94% in Sweden [17,18,19]. It is difficult to say how comparable the results are because of the variation in sampling techniques and the frequency of sampling. In addition, in our study, we did not look at hydraulic retention time which is necessary when comparing the influent and the effluent. However, it could be expected that in LMICs, where resources are limited, wastewater treatment plants might be less up-to-date and function less effectively than in high income countries.

Per month, 34 µg/L of the studied antibiotics from the rural hospital and 32.4 µg/L from the urban hospital were released into the environment. The amount of water used in the rural hospital was approximately 1800 m^3^ per month [personal communication with the hospital staff in charge of the wastewater treatment plant], meaning that every month about 61 g of the studied antibiotics were released into the environment from the hospital. Among the studied antibiotics, CIP was detected at the highest concentrations in the rural hospital. CIP, a second generation fluroquinolone, was also found at high concentrations in hospital effluents in various studies [20,21,22,23,24]. Its high residues might be explained in two ways: that it is one of the most prescribed antibiotics in hospitals and it is not readily biodegradable [25]. CIP at a residue levels as low as 25 µg/L can cause modification in bacterial strains and cause genotoxic effects [26]. In the present study, CIP was found in wastewater after treatment with concentration ranging up to 53.3 µg/L and this relatively high concentration is a matter of concern. In the urban hospital, the antibiotic detected at highest concentration was MET. MET is an antibiotic widely used to treat infectious diseases caused by anaerobic bacteria and protozoa. The high solubility of MET in water and its low biodegradability makes it a difficult contaminant to remove by traditional methods of wastewater treatment, causing its accumulation in the aquatic environment [27]. CEF residue was detected only in two months in the rural hospital and was not detected in any of the samples in the urban hospital. Neither CEF nor other cephalosporins have been detected in hospital effluents elsewhere. The reasons for their non-detection or very rare detection can be the easy degradation of the β-lactam ring, its high metabolic rate and the process of decarboxylation [1].

Logically, it can be presumed that residue levels of antibiotics in hospital wastewater will depend on the quantities of antibiotics used in the hospitals. When comparing the measured concentrations for different active pharmaceutical ingredients including antibiotics in hospital wastewater with the predicted concentrations based on drug consumption data of the hospital, Daouk *et al.* found a ‘good accuracy’ for MET, SUL, and CIP [28]. In the present study, no antibiotics were detected in incoming water in both the hospitals, meaning that antibiotic residues in hospital wastewater, if any, were from the hospitals’ use. Significant correlations were found between the quantities of antibiotics used and antibiotic concentrations in wastewater before treatment of the rural hospital for MET and CIP. These two antibiotics had low biodegradability and were detected at the highest concentrations in the hospital. In the case of other antibiotics, there could be other factors that can play roles in antibiotic residue level. For example: the metabolism of antibiotics and their stability in the environment. OFL was detected in the lowest concentrations in wastewater in the rural hospital although it was not on the antibiotic use data. It is possible that patients might have used the antibiotic before admission or it might have been purchased from outside and used while admitted in the hospital. It has not been established beyond doubt, which is the correct method to find out correlation between antibiotic consumption in a hospital and the antibiotic concentration in the wastewater of the same hospital. Efforts have been made to indicate predicted environmental concentrations from the annual data of hospital antibiotic consumption [29]. In our effort to analyze correlation with the hospital consumption, we could only get monthly antibiotic consumption data from the hospital. Measuring antibiotic concentration daily was beyond our financial means. Therefore, 24 h measured antibiotic residue concentrations in the last week of the month were considered representative of a month. We found that the correlation between measured antibiotic concentrations in hospital wastewater in a month and the monthly antibiotic consumption or the calculated daily average consumption of antibiotics of that month remained the same irrespective of comparing the concentration.

There has been a growing concern that the presence of antibiotics in the environment can contribute to the development of antibiotic resistance in bacteria [30]. It was indicated by Kummerer *et al.* that the predicted antibiotic concentrations in the hospital effluent might exceed the minimum inhibitory concentrations of susceptible pathogenic bacteria, leading to a selection pressure on the bacteria [29]. Significant correlations between the concentration of antibiotic residues and the prevalence of antibiotic resistant bacteria were observed in wastewater treatment plants [31,32]. It is still unclear to what extent this contribution is, however, it is well-known that with very low antibiotic concentrations below the minimum inhibitory concentrations (MICs), the selection of resistant bacteria can occur [33,34,35]. Predicted no-effect concentrations (PNECs) of antibiotics for resistance selection were reported in one study, where PNEC for MET, SUL, TRI, CEF, CIP, OFL, and SPI were 0.125 µg/L; 16 µg/L; 0.5 µg/L; 0.5 µg/L; 0.064 µg/L; 0.5 µg/L; and 0.5 µg/L, respectively [36]. The minimum selective concentration (MSC) for CIP was reported to be 0.1 µg/L in another study [33]. Antibiotic concentrations in the hospital wastewater from our study in many cases were higher than the reported PNECs and MSC. Thus, the selection of antibiotic resistant bacteria can occur. From the hospital effluents, antibiotic residues and antibiotic resistant bacteria can be released to the environment. They may enter the water bodies, from which water may be used for irrigation or for household purposes, possibly resulting in conditions that affect infections by resistant bacteria in humans and animals.

Antibiotic resistance has become a serious and growing public health threat worldwide. Due to that, in many areas of the world, there are few or even no effective antibiotic therapies available for life-threatening infections [37]. In 2015, the 68th World Health Assembly endorsed a global action plan, addressing all stakeholders, to tackle antimicrobial resistance, focusing antibiotic resistance [38]. A recent review shows, e.g., that the public have an incomplete understanding of antibiotic resistance and misperceptions about it and its causes [39]. It emerged from our findings that hospital antibiotic use resulted in the presence of antibiotics in hospital wastewater at concentrations that can promote the selection of antibiotic resistant bacteria. A further study is suggested to determine antibiotic resistance in bacteria isolates from wastewater of the hospitals.

Various studies indicated that wastewater treatment plants are considered as “hotspots” for the release of antibiotics and also antibiotic resistant bacteria into the environment [40,41,42]. In LMICs, where many wastewater treatment plants function ineffectively, these ‘hotspots’ could play an even more significant role in releasing antibiotics and in the selection for antibiotic resistant bacteria. In Vietnam, almost 50% of hospitals across the country still do not have wastewater treatment plants [14]. Therefore, from the point of view of LMICs, there is a need for new and effective hospital wastewater treatment plants for the removal of antibiotic residues.

This is one of few studies globally which has attempted to identify concurrently antibiotic residues in hospital wastewater and its relationship with antibiotic use in the same hospital. Continuous sampling method is considered a method of choice for determining concentrations of pharmaceuticals in hospital wastewater. In the present study, we used continuous constant sampling with a pump of a fixed speed because that was the only feasible thing under the conditions existing at that time of conducting the study. A continuous constant sampling method has shown less sampling uncertainty than grab sampling. However, to minimize the sampling error, continuous flow-proportional sampling mode would be the optimal method [43,44]. A limitation of the study is that samples could not be collected during three months from June to August in the urban hospital due to the reconstruction of the hospital. Another limitation is that a detailed description of the wastewater treatment plants was not available to us. The lower antibiotic concentrations after wastewater treatment compared to before treatment was relatively comparable to some studies from high income countries. We believe that the results of this study can probably be transferred to other settings with relatively similar contexts and will contribute to further scientific enquiry.

## 5. Conclusions

Although there were lower antibiotic concentrations after wastewater treatment, still there were significant amounts of antibiotics present in the wastewater of both the rural and the urban hospital. Significant correlations were found between the quantities of antibiotics used and antibiotic concentrations in wastewater before treatment of the rural hospital for ciprofloxacin and metronidazole. The hospital antibiotic use contributed to the release of antibiotics into the environment, which can promote the development of antibiotic resistance.

## Figures and Tables

**Table 1:** Total concentrations (µg/L) of studied antibiotics in wastewater of a rural and an urban hospital and the total quantities of studied antibiotics used in the rural hospital in Vietnam.

Month	Rural Hospital				Urban Hospital		
	Quantity of Studied ABs Used (g)	WBT (µg/L)	WAT (µg/L)	Difference (%)	WBT (µg/L)	WAT (µg/L)	Difference (%)
January	398.2	28.8	13.8	52.0	18.0	12.4	30.9
February	215.3	29.2	7.8	73.2	8.7	2.4	72.3
March	669.8	75.2	48.2	36.0	17.9	8.7	51.7
April	552.5	106.9	25.9	75.8	39.9	10.8	72.9
May	590.5	21.8	10.3	52.8	29.1	6.0	79.4
June	852.4	22.0	16.5	25.1	N	N	N
July	842.3	30.0	20.1	32.9	N	N	N
August	815.7	58.5	46.0	21.4	N	N	N
September	539.7	133.5	82.2	38.4	151.1	14.2	90.6
October	877.5	124.2	33.1	73.3	202.3	40.3	80.1
November	875.5	131.8	71.7	45.6	37.4	5.6	85.0
December	630.0	83.5	32.3	61.3	337.3	191.3	43.3
**Mean**	**654.9**	**70.5**	**34.0**	**49.0**	**93.5**	**32.4**	**67.3**
**Mean per bed**	**3.0**	**0.32**	**0.15**		**0.18**	**0.06**	

WBT: Wastewater before treatment; WAT: Wastewater after treatment; ABs: antibiotics; N: No data.

**Table 2:** Concentrations (µg/L) of studied antibiotics in wastewater of a rural hospital in Vietnam.

Month	MET *		SUL		TRI *		CEF		CIP *		OFL *		SPI *	
	WBT	WAT	WBT	WAT	WBT	WAT	WBT	WAT	WBT	WAT	WBT	WAT	WBT	WAT
January	0.1	0.1	1.9	1.9	3.7	0.7	-	-	18.0	9.8	3.1	0.8	2.0	0.5
February	-	-	0.4	0.2	1.2	0.1	-	-	26.0	6.4	1.2	1.1	0.4	-
March	0.2	-	2.7	2.9	4.3	0.5	11.0	5.0	56.4	39.5	-	-	0.7	0.4
April	0.2	0.2	12.2	3.7	15.5	0.8	2.8	2.6	66.0	16.2	9.4	2.0	0.8	0.2
May	0.2	-	3.2	3.8	3.0	0.5	-	-	7.9	5.6	7.1	-	0.5	0.4
June	1.2	-	1.2	4.5	1.4	0.7	-	-	17.4	11.0	-	-	0.9	0.3
July	2.1	0.2	3.7	3.4	1.8	0.5	-	-	16.1	13.9	2.0	-	4.3	2.2
August	-	-	23.5	20.3	5.7	4.3	-	-	26.3	19.5	3.1	2.0	-	-
September	19.9	16.4	17.2	11.0	2.8	0.9	-	-	79.9	48.1	5.3	4.6	8.4	1.2
October	-	-	25.5	11.2	19.1	5.0	-	-	71.5	16.3	5.2	-	2.9	0.6
November	6.3	-	17.5	8.3	10.9	2.7	-	-	87.3	53.3	9.7	7.4	-	-
December	1.0	-	9.1	5.4	22.4	1.1	-	-	41.3	18.7	9.7	7.1	-	-
**Total**	**31.3**	**16.9**	**118.0**	**76.5**	**91.8**	**17.9**	**13.8**	**7.6**	**514.0**	**258.2**	**55.7**	**25.1**	**20.9**	**5.8**
**Mean**	**2.6**	**1.4**	**9.8**	**6.4**	**7.7**	**1.5**	**1.2**	**0.6**	**42.8**	**21.5**	**4.6**	**2.1**	**1.7**	**0.5**
**Average Difference**		**46.0%**		**35.2%**		**80.5%**		**45.0%**		**49.8%**		**54.9%**		**72.3%**

WBT: Wastewater before treatment; WAT: Wastewater after treatment; “-“: Below Limit of Detection; * Differences between mean values of antibiotic concentrations before and after wastewater treatment are significant (*p* values are presented below with antibiotic names); MET: Metronidazole (*p* = 0.01); SUL: Sulfamethoxazole (*p* = 0.06); TRI: Trimethoprim (*p* = 0.002); CEF: Ceftazidime (*p* = 0.16); CIP: Ciprofloxacin (*p* = 0.002); OFL: Ofloxacin (*p* = 0.003); SPI: Spiramycin (*p* = 0.004).

**Table 3:** Concentrations (µg/L) of studied antibiotics in wastewater of an urban hospital in Vietnam.

Month	MET *		SUL *		TRI *		CEF		CIP *		OFL *		SPI *	
	WBT	WAT	WBT	WAT	WBT	WAT	WBT	WAT	WBT	WAT	WBT	WAT	WBT	WAT
January	0.3	0.1	0.8	1.2	0.6	0.7	-	-	7.5	3.1	6.9	5.7	1.9	1.7
February	-	-	0.2	0.1	0.2	0.1	-	-	1.7	0.6	2.9	1.6	3.6	-
March	0.8	-	0.9	0.3	0.4	0.2	-	-	15.7	8.0	-	-	0.2	0.2
April	0.6	0.2	0.8	0.1	5.4	0.4	-	-	3.6	1.4	22.8	7.3	6.7	1.5
May	0.2	0.2	2.7	0.1	1.9	0.4	-	-	7.0	0.8	17.1	4.3	0.3	0.4
June	N	N	N	N	N	N	N	N	N	N	N	N	N	N
July	N	N	N	N	N	N	N	N	N	N	N	N	N	N
August	N	N	N	N	N	N	N	N	N	N	N	N	N	N
September	64.0	2.6	31.4	4.5	2.0	0.8	-	-	30.2	4.1	21.8	2.3	1.7	-
October	-	-	19.1	18.9	8.0	7.1	-	-	60.3	10.3	111.1	4.1	3.7	-
November	4.0	-	16.0	1.5	3.2	1.6	-	-	5.3	2.6	8.9	-	-	-
December	258.3	130.4	15.3	-	2.7	0.9	-	-	40.4	40.2	20.7	19.8	-	-
**Total**	**328.2**	**133.5**	**87.2**	**26.5**	**24.3**	**11.9**	**-**	**-**	**171.8**	**71.1**	**212.2**	**45.1**	**18.1**	**3.7**
**Mean**	**36.5**	**14.8**	**9.7**	**3.0**	**2.7**	**1.3**	**-**	**-**	**19.1**	**7.9**	**23.6**	**5.0**	**2.0**	**0.4**
**Average Difference**		**59.3%**		**69.6%**		**51.3%**		**-**		**58.6%**		**78.7%**		**79.6%**

WBT: Wastewater before treatment; WAT: Wastewater after treatment; “-“: Below Limit of Detection; N: No data; * Differences between mean values of antibiotic concentrations before and after wastewater treatment are significant (*p* values are presented below with antibiotic names); MET: Metronidazole (*p* = 0.03); SUL: Sulfamethoxazole (*p* = 0.02); TRI: Trimethoprim (*p* = 0.01); CEF: Ceftazidime; CIP: Ciprofloxacin (*p* = 0.008); OFL: Ofloxacin (*p* = 0.009); SPI: Spiramycin (*p* = 0.04).

## Abbreviations

The following abbreviations are used in this manuscript:

LMICs	low- and lower middle-income countries
MET	Metronidazole
SUL	Sulfamethoxazole
TRI	Trimethoprim
CEF	Ceftazidime
CIP	Ciprofloxacin
OFL	Ofloxacin
SPI	Spiramycin
WBT	Wastewater before treatment
WAT	Wastewater after treatment
µg/L	Microgram per liter
